# Analysis of the *VSX1* gene in sporadic keratoconus patients from China

**DOI:** 10.1186/s12886-017-0567-3

**Published:** 2017-09-26

**Authors:** Tao Guan, Xue Wang, Li-Bin Zheng, Hai-Jian Wu, Yu-Feng Yao

**Affiliations:** 1 Department of Ophthalmology, Taizhou Municipal Hospital, Taizhou, 318000 China; 2 Laboratory of Kidney Carcinoma, The Second Hospital of Ningbo Yinzhou, Ningbo, 315000 China; 3 Department of Ophthalmology, Sir Run Run Shaw Hospital, Zhejiang University School of Medicine, Hangzhou, 310016 China

**Keywords:** Sporadic keratoconus, *VSX1* gene, Sequence variant, Single-nucleotide polymorphism

## Abstract

**Background:**

Keratoconus normally presents as a sporadic disease. Although different studies have found sequence variants of the visual system homeobox 1 (*VSX1*) gene associated with keratoconus in humans, no research has detected such variants in sporadic keratoconus patients from China. To investigate the possibility of *VSX1* being a candidate susceptibility gene for Chinese patients with sporadic keratoconus, we performed sequence screening of this gene in such patients.

**Methods:**

Whole DNA was obtained from the leukocytes in the peripheral venous blood of 50 patients with sporadic keratoconus and 50 control subjects without this ocular disorder. Polymerase chain reaction single-strand conformation polymorphism analysis and direct DNA sequencing technology were used to detect sequence variation in the five exons and splicing regions of the introns of the *VSX1* gene. The sequencing results were analyzed using DNAstar software.

**Results:**

One novel missense heterozygous sequence variant (p.Arg131Pro) was found in the first exon of the *VSX1* gene in one keratoconus patient. Another heterozygous sequence variant (p.Gly160Val) in the second exon was found in two keratoconus patients. These variants were not detected in the control subjects. In the third intron of the *VSX1* gene, c.8326G > A nucleotide substitution (including heterozygous and homozygous change) was also discovered. The frequency of this variation did not differ significantly between patients and controls, it should belong to single-nucleotide polymorphism of the *VSX1* gene. Bioinformatic analysis also predicted that one missense sequence variation (p.Arg131Pro) may not cause a pathogenic change.

**Conclusions:**

In this study, we added one novel missense sequence variation (p.Arg131Pro) in the coding region of the *VSX1* gene to the range of *VSX1* coding region variations observed in patients with sporadic keratoconus from China. Our work suggests that *VSX1* sequence variants might be involved in the pathogenesis of sporadic keratoconus, but their precise role in disease causation requires further investigation.

## Background

Keratoconus is a congenital disease involving noninflammatory corneal ectasia. Its prevalence is estimated to be 1/2000 in the general population, and the incidence ratio between males and females is 1.7:1 [1, 2]. Although most keratoconus cases are sporadic, about 6–10% of cases have been reported to have a positive family history. Keratoconus can exhibit dominant or recessive inheritance; with autosomal dominant inheritance, the disease exhibits variable phenotypes with incomplete penetrance [3, 4]. The pathological features of keratoconus include decreased corneal collagen or the abnormal distribution of collagen fiber, which reduce the mechanical resistance of the cornea and therefore cause the center of the cornea to protrude, leading to a thin conical shape. Slit lamp microscope examinations of keratoconus patients have demonstrated Vogt’s line, Fleischer’s ring, and corneal scarring [5–7]. The onset of the disease occurs during puberty, involving both eyes, and with highly irregular astigmatism. In the late phase, patients show acute corneal edema and scar formation, along with significant vision loss. Although several treatment modalities are available, severe keratoconus remains an indication for corneal transplantation [8–10].

Genome-wide linkage analyses have identified several chromosomal loci and genes that may be related to keratoconus; some of these were eventually ruled out, while for others, an association with this disease could not be confirmed. One of the candidate genes strongly implicated in keratoconus is visual system homeobox gene 1 (*VSX1*) localized to chromosome 20 p11–q11 [11–14]. The *VSX1* gene encodes a paired-like homeodomain transcription factor. It is associated with eye development and was first isolated and cloned from a cDNA library of the retina of adult goldfish. In the human body, the *VSX1* gene is expressed in the embryonic craniofacial region, the granular layer of adult retina, and corneal tissue. The human *VSX1* gene has five exons and encodes a 365-amino-acid protein with a homeobox DNA binding domain and a CVC domain, being highly conserved among vertebrates. Barbaco et al. [15] reported that the *VSX1* gene was activated in corneal stroma during the process of corneal wound repair in vitro and in vivo, accompanied by the transformation of corneal stromal cells into myofibroblasts. These results implied that *VSX1* gene variants may play an important role in the development of keratoconus. Several variants of the *VSX1* gene [16–19] have been reported from various parts of the world, but a definitive pathogenic role of these variants in keratoconus has not yet been established because segregation of these variants was also seen in some unaffected individuals.

To our knowledge, this is the first study to perform screening for variants of the *VSX1* gene in the Chinese population and to explore their role in keratoconus. Nucleotide variations in the five exons and the splicing regions of introns of the *VSX1* gene were examined by polymerase chain reaction single-strand conformation polymorphism (PCR-SSCP) analysis and direct DNA sequencing in 50 Chinese patients with keratoconus, which were compared with the sequences in 50 controls.

## Methods

### Subjects and clinical examination

All sporadic keratoconus patients and control subjects were identified at the Department of Ophthalmology, Taizhou Municipal Hospital. A total of 50 patients were diagnosed with sporadic keratoconus during the period of March 2013 to July 2015, including 29 males and 21 females, aged from 15 to 35 years old. The diagnosis was based on symptoms and the results of slit lamp microscope examinations; the diagnostic features included progressive curvature of the anterior corneal surface steepening, corneal thinning in the same region, Fleischer’s ring, and Vogt’s stripes, combined with the corneal surface topography and a caudal surface of the cornea thicker than 20 μm. Patients were considered as sporadic cases after examining the immediate family members and identifying the patient as an isolated case of keratoconus. Another 50 healthy people who underwent detailed ocular and ophthalmological examinations that ruled out any ophthalmic diseases were selected as a control group to determine whether the detected variation was SNP of *VSX1* gene. The control subjects included 31 males and 19 females, aged from 20 to 36 years old. The age and gender generally matched between the two groups and systemic physical examinations were performed in both groups to rule out other concurrent diseases. This study followed the Declaration of Helsinki and was reviewed by the ethics committee of Taizhou Municipal Hospital. Written informed consent was obtained from all of the subjects in this study.

### Genetic study

Three milliliters of peripheral venous blood was extracted from the subjects and stored in EDTA anticoagulation tubes. Genomic DNA was isolated from blood leukocytes using the Dzup (Blood) Genomic DNA Isolation Reagent (B518205; Sangon Biotech Co., Ltd., Shanghai, China), in accordance with the manufacturer’s standard methods.

Based on previous studies [20, 21] and the DNA sequence of the human *VSX1* gene from the NCBI database, primers were designed for the five exons of this gene. These primers were synthesized by Sangon Biotech Co., Ltd. (Shanghai, China). Sequences of the primers are listed in Table 1.

**Table 1:** List of primers and PCR amplification conditions used for *VSX1* gene amplification

Exon	Sequence of primers (5′-3′)	Annealing temperature (°C)	Product length (bp)
1	CAGCTGATTGGAGCCCTTC(sense)	62	599
	CTCAGAGCCTAGGGGACAGG(antisense)		
2	CTTAAGTACCCAAGAGGTTCATAACT(sense)	62	269
	GAAACCACTGGGCCTGCTATCAT(antisense)		
3	AAGCAGGCACGGTGGTCCTTA(sense)	63	293
	AAGGGACTGCTGATTGGCTCACT(antisense)		
4	GCTCGGGAGAGAAGATCC(sense)	62	399
	TCAGTAAACTGACGTTGCTTG(antisense)		
5	GGAAATTTACTTCATTGCTGAATTT(sense)	60	495
	TGGCATTGCATTTTATCTTGACA(antisense)		

The HBP 220 PCR cycler (HYBAID, England) was used for a 50-μL PCR reaction system containing 200 ng of DNA template, 5 μL of 10× buffer solution, 1.5 mmol/L MgCl_2_, 0.25 μmol/L primers, 100 μmol/L of each dNTP, and 2 U of Taq DNA polymerase (Sangon Biotech Co., Ltd., Shanghai, China). The PCR procedure was as follows: 94 °C for 5 min; 35 amplification cycles of denaturation at 94 °C for 45 s, annealing for 45 s, and extension at 72 °C for 50 s; and finally extension at 72 °C for 10 min. PCR products were examined by 2% agarose gel electrophoresis and EB (Ethidium Bromide) staining. The annealing temperatures of each exon and the fragment sizes of PCR products are listed in Table 1.

An 8% (49:1) nondenaturing polyacrylamide gel with 5% glycerol was used for SSCP analysis and silver staining. Here, 5-μL PCR products mixed with sample buffer (90% formamide, 0.05% bromophenol blue, and 0.05% xylene blue) were processed for denaturation at 95 °C for 10 min, placed in an ice bath, and then subjected to electrophoresis (100 V) at room temperature for 5 to 6 h (when xylene blue reached the bottom of the gel). Then, the gel was fixed in 10% ethanol for 5 min and soaked in 1% silver nitrate for 5 min, followed by staining in 0.012 mol/L silver nitrate and 0.05% formaldehyde for 10 min. Next, the gel was processed in 0.28 mol/L sodium carbonate, 0.1% formaldehyde, and 0.1 g/L thiosulfate sodium for colorization, which was stopped by immersion in 10% acetic acid.

DNA with an abnormal banding pattern in electrophoresis was detected by SSCP and silver staining analysis. Its PCR product was then purified using isopropyl alcohol and bidirectionally screened to identify the sequence variant using the ABI3730XL DNA Analyzer (Sangon Biotech Co., Ltd., Shanghai, China). Nucleotide sequences were then compared with the published *VSX1* cDNA sequence (GenBank NM_014588).

### Bioinformatic analysis

The in silico prediction programs Polyphen-2 (http://genetics.bwh.harvard.edu/pph2/) and PROVEAN (http://provean.jcvi.org/index.php) were used to predict the effect of the *VSX1* gene sequence variants on protein structure and function [22–24].

### Statistics

To determine statistically significant differences between the groups, data analysis was performed using SPSS version 18.0. Continuous data are displayed here as mean ± standard deviation. Differences between two groups were examined by *t*-test. Repeated measure ANOVA was used to analyze the differences among multiple groups. Values of *P* < 0.05 were considered statistically significant.

## Results

### PCR-SSCP results

**Fig. 1:**
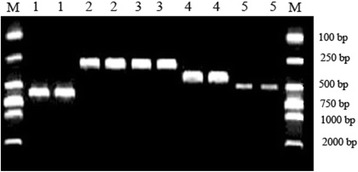
Electrophoresis pattern of PCR products of the *VSX1* gene. M: DNA markers, 1: first exon (599 bp), 2: second exon (269 bp), 3: third exon (293 bp), 4: fourth exon (399 bp), 5: fifth exon (495 bp)

All exons of the *VSX1* gene in sporadic keratoconus patients were amplified by PCR. The PCR products showed high specificity and efficiency in amplification by 2% agarose gel electrophoresis, with a single band corresponding to the correct fragment length (Fig. 1). This suggested that there were no sequence variants with a large insertion or deletion in the five exons of the *VSX1* gene in the sporadic keratoconus patients. By SSCP analysis and silver staining, four types of abnormal banding pattern were clearly shown in the electrophoresis gels, suggesting the presence of four sequence variants in the five exons of the *VSX1* gene.

### DNA sequencing results

The 50 sporadic keratoconus patients and 50 control subjects recruited for this study underwent screening of the entire coding region and exon–intron junctions of the *VSX1* gene by direct sequencing of DNA. The DNA sequencing results confirmed the abnormal banding patterns found in the SSCP analysis and silver staining as described above. The results revealed four nucleotide sequence variants of the *VSX1* gene in Chinese sporadic keratoconus patients, including two missense sequence variants and one SNP change (heterozygous and homozygous) (Table 2).

**Table 2:** List of sequence variants of the *VSX1* gene in Chinese sporadic keratoconus patients

Nucleotide change	Amino acid change	Change site	Change type	Keratoconus (*n* = 50)	Controls (*n* = 50)
c.5427G > C	p.Arg131Pro	First exon	Heterozygous missense variant	1 (2.0%)	0 (0.0%)
c.7672G > T	p.Gly160Val	Second exon	Heterozygous missense variant	2 (4.0%)	0 (0.0%)
c.8326G > A	Noncoding region	Third intron	Heterozygous SNP variant	3 (6.0%)	4 (8.0%)
c.8326G > A	Noncoding region	Third intron	Homozygous SNP variant	2 (4.0%)	1 (2.0%)

**Fig. 2:**
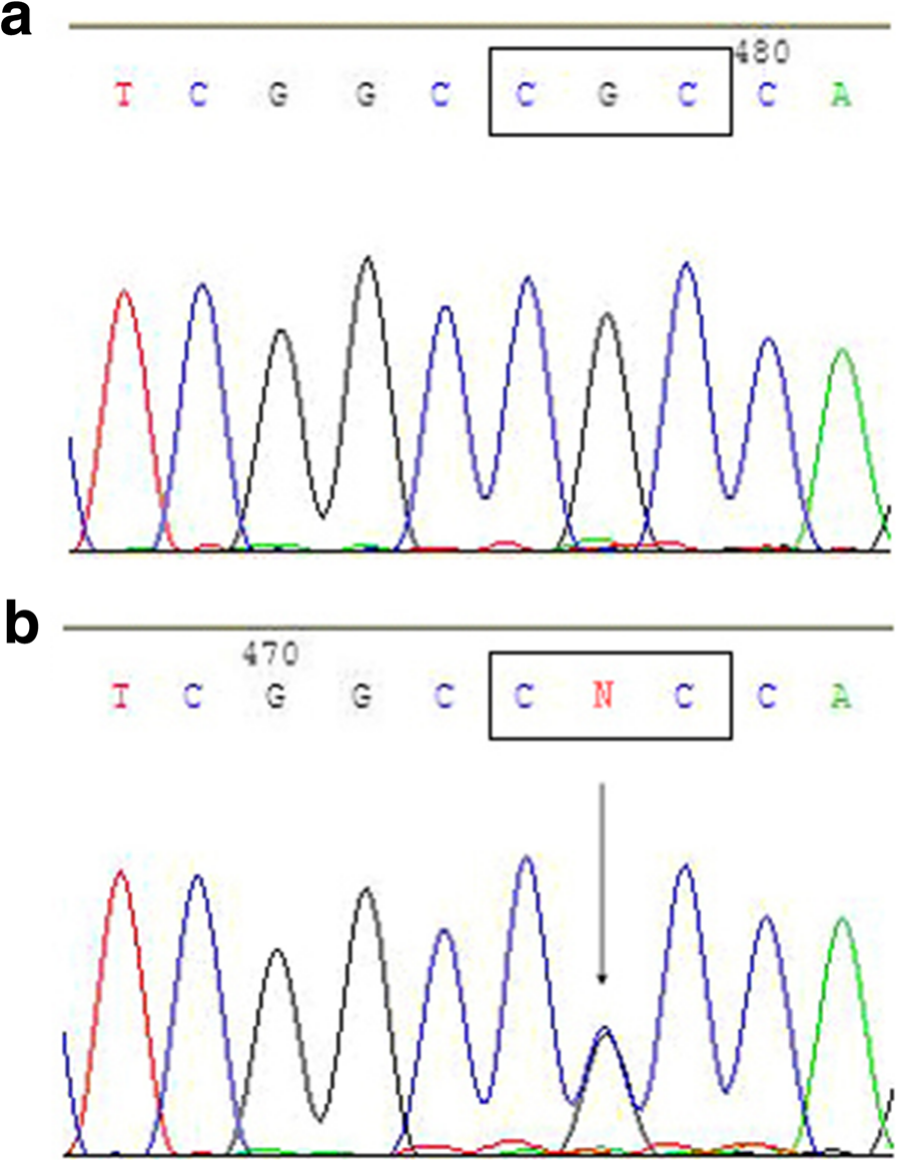
The p.Arg131Pro variant of the *VSX1* gene. The sequence of a normal control (**a**) included the nucleotides CGC at codon 131 in exon 1 of *VSX1*. The missense variant sequence (**b**) showed a G to C (c. 5427 G > C) heterozygous transition at the second nucleotide position in this codon (arrow), which results in a change from arginine (CGC) to proline (CCC) (p.Arg131Pro)

The sequence variant c.5427G > C was found in the first exon of the *VSX1* gene in one case of sporadic keratoconus. This involved a change in the codon from CGC to CCC, leading to a missense substitution of arginine to proline at codon 131 (p.Arg131Pro sequence variant). p.Arg131Pro was not detected in any control subjects (Fig. 2) and had not previously been identified. We registered it in a public database at https://www.ncbi.nlm.nih.gov/projects/SNP/ (ID: rs267597889). Using the PolyPhen-2 tool, p.Arg131Pro was assessed as being benign, in terms of its phenotypic effects, with a score of 0.344; using the PROVEAN tool, it was assessed as being neutral, with a score of −0.048. Both tools predicted that the replaced amino acid would have no adverse effects and would be tolerated.

**Fig. 3:**
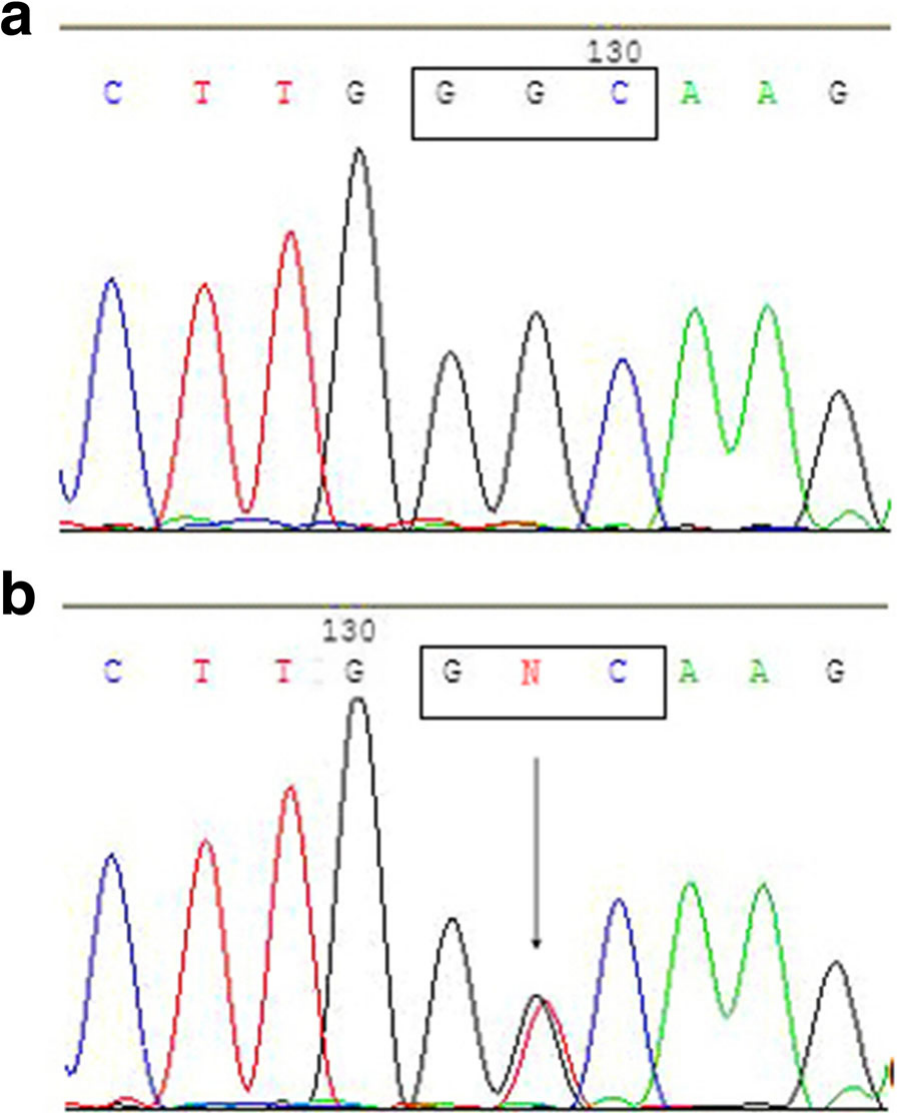
The p.Gly160Val variant of the *VSX1* gene. The sequence of a normal control (**a**) included the nucleotides GGC at codon 160 in the second exon of *VSX1*. The missense variant sequence (**b**) showed a G to T (c. 7672 G > T) heterozygous transition at the second nucleotide position in this codon (arrow), which results in a change from glycine (GGC) to valine (GTC) (p.Gly160Val)

The other missense sequence variant c.7672G > T was found in the second exon of the *VSX1* gene in two cases of sporadic keratoconus. This involves a change in codon 160 from GGC to GTC, leading to a missense substitution of glycine to valine (p.Gly160Val sequence variant) (Fig. 3). No similar change was found in any of the 50 normal controls.

**Fig. 4:**
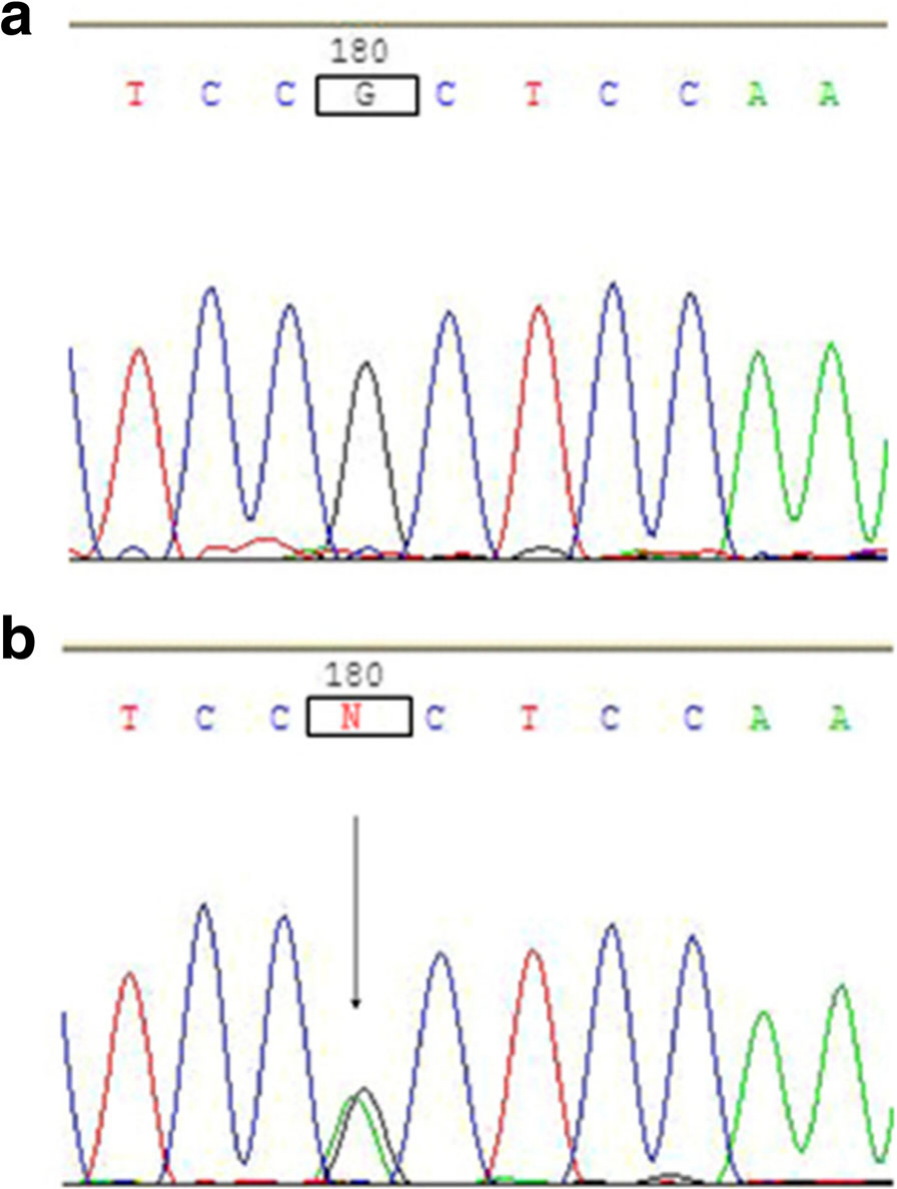
The c.8326G > A heterozygous change of the *VSX1* gene. The sequence of a normal control (**a**) included the nucleotide G at position 8326 in the third intron of *VSX1*. The variant sequence (**b**) showed a G to A heterozygous transition (arrow), which results in a c.8326G > A SNP change

**Fig. 5:**
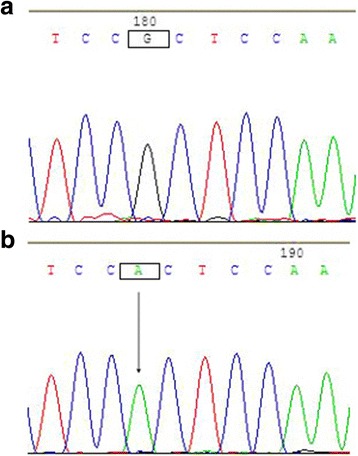
The c.8326G > A homozygous change of the *VSX1* gene. The sequence of a normal control (**a**) included the nucleotide G at position 8326 in the third intron of *VSX1*. The variant sequence (**b**) showed a G to A homozygous transition (arrow), which results in a c.8326G > A SNP change

In the third intron of the *VSX1* gene, the substitution of G > A at nucleotide position 8326 (c.8326G > A) was identified, which was present in heterozygous form in three cases of sporadic keratoconus and in four controls (Fig. 4). It was also present in homozygous form in two sporadic keratoconus patients and one control (Fig. 5). There was no significant difference in the incidence of c.8326G > A between the two groups.

## Discussion

Keratoconus is a complex condition with a multi-factorial etiology that normally occurs as a sporadic disorder [25]. Recent studies have revealed the involvement of various genes in its development, such as *VSX1*, *SOD1*, *COL6A1*, *TGFBI*, *DOCK9*, *STK24*, and *IPO5* [26–28]. Despite elucidate the etiology and the disease progression were conducted by numerous research, *VSX1* is the only gene indicated as a vital genetic factor in determining the keratoconus [29]. Verma et al. ruled out the involvement of the *VSX1* gene in South Indian patients with sporadic keratoconus [30]. However, Shetty et al. found that p.Leu268His, a novel missense variation in the coding region of VSX1, might be involved in the pathogenesis of sporadic keratoconus in Indians [22]. These earlier studies indicated the need for further study on the relationship between *VSX1* pathogenic variations and sporadic keratoconus in different subsets of the keratoconus-affected population. Shetty et al. also summarized the *VSX1* coding variants that are associated with keratoconus in different ethnic groups, but the Chinese were not included in this work (Table 3) [16–19, 22, 29, 31–38]. In the present study, PCR-SSCP and direct DNA sequencing techniques were used to examine nucleotide variations in the five exons and splicing regions of introns of the *VSX1* gene in 50 unrelated Chinese patients with keratoconus, which were compared with the sequences in 50 controls. The results showed the presence of four nucleotide variations in sporadic keratoconus patients, consisting of a missense p.Arg131Pro sequence variant (heterozygous), a missense p.Gly160Val sequence variant (heterozygous), and nucleotide c.8326G > A sequence variation (in both heterozygous and homozygous forms). p.Gly160Val was located in the second exon of the *VSX1* gene. In 2008, Mok et al. [33] first reported that p.Gly160Val variation was found in 13 out of 249 cases (5.3%) of sporadic keratoconus in South Korea, being located in the homeobox DNA binding domain of *VSX1* gene coding regions and involving a polar amino acid being replaced by a neutral one. This may affect the binding of the *VSX1* protein to DNA and modify the transcription rate. Therefore, it was believed that p.Gly160Val of the *VSX1* gene increases the risk of the onset of keratoconus. In this study, we identified p.Gly160Val in two cases of 50 sporadic keratoconus patients, the sequence variant rate was 4.0%, but was absent in the control group. The findings suggested that p.Gly160Val may be the cause of two cases of sporadic keratoconus among these Chinese subjects. However, further biophysical studies are necessary to evaluate the precise molecular mechanism behind the effects of this variant form of *VSX1*.

**Table 3:** Summary of *VSX1* coding variants identified in patients with keratoconus [22]

Coding variants	Clinical significance	Phenotype	Unrelated Controls	Ethnic groups	References
p.Leu 17 Pro	Pathogenic^a^	Keratoconus	–	Italian	[17]
p.Leu 17 Val	Nonpathogenic	Keratoconus	+	Korean	[31]
p. Pro 58 Leu	Pathogenic^a, b^	Keratoconus	–	Caucasian	[32]
p. Leu 159 Met	Unknown	Keratoconus	–	Caucasian	[16, 18]
p. Asn 151 Ser	Pathogenic^a^	Keratoconus	–	Korean	[33]
p. Gly 160 Val	Nonpathogenic	Keratoconus	+	Korean	[31, 33]
p. Val 199 Leu	Nonpathogenic	Keratoconus	+	Korean	[31]
p. Arg 166 Trp	Unknown	Keratoconus	+	Caucasian, Iranian	[16, 34]
p. Gln 175 His	Unknown	Keratoconus	–	Indian	[35]
p. Arg 217 His	Nonpathogenic	Keratoconus	+	Indian, Pakistan, European	[19, 36]
p. Gly 239 Arg	Pathogenic^a, b^	Keratoconus	–	Italian	[29]
p. His 244 Arg	Unknown	Keratoconus	+	Caucasian, Iranian	[16, 18, 34, 37]
p. Ser 251 Thr	Unknown	Keratoconus	–	Indian	[22]
p. Pro 247 Arg	Nonpathogenic	Keratoconus	+	Italian	[16, 17, 38]
p. Leu 268 His	Pathogenic^a, b^	Keratoconus	–	Indian	[22]

Coding variants of the VSX1 gene have been reported in studies based on an original report^a^ and bioinformatic predictions^b^. The symbols “+” and “−” represent present and absent, respectively

The c.8326G > A sequence variation occurred in the third intron of the *VSX1* gene. Abu-Amero et al. [25] detected c.8326G > A heterozygous variation in the Saudi Arabian population. They identified this variation in keratoconus patients as well as in controls, with no difference in its incidence between the two groups. This variation is located within an intron and does not cause a change in the amino acid sequence encoded by the *VXS1* gene. Therefore, they considered that c.8326G > A variation belonged to SNP change of *VSX1* gene. In our study, the c.8326G > A variant of the *VSX1* gene was identified in both heterozygous and homozygous form, and it did not differ significantly in its incidence between the two groups. Overall, our results demonstrate the existence of SNPs in the *VSX1* gene in the Chinese population, and suggest that sporadic keratoconus patients from different ethnic groups may have the same pathogenesis.

In the present study, we identified one novel heterozygous variant c.5427G > C (p.Arg131Pro; rs267597889) of the *VSX1* gene in a 20-year-old affected male, which was absent from all 50 controls. This patient had a history of keratoconus lasting more than 5 years. The slit lamp microscope examination showed conical protrusion (opacity at the top) of the cornea in both eyes, with corneal thinning and a vertical linear scar, as well as complete Fleischer’s ring. p.Arg131Pro (replacing the amino acid arginine with proline) has not been reported previously, so this work adds to the range of variants in the *VSX1* gene and suggests the role of this novel variant in keratoconus. However, predictions made using the PolyPhen-2 and PROVEAN tools suggested that this variant would not have detrimental phenotypic effects. This is explained by the fact that the 131st amino acid arginine is located in a noncritical open reading frame region of the *VSX1* gene, so its character is not particularly important and it has not been conserved among the gene’s orthologs. The presence of p.Arg131Pro in just 2% of the patients in this study reinforces the assertion that this is a minor gene, but its exact role during development needs to be clarified.

## Conclusions

In summary, the p.Arg131Pro, p.Gly160Val, and c.8326G > A variations in the *VSX1* gene were identified for the first time in Chinese patients with sporadic keratoconus. We added one novel missense sequence variation (p.Arg131Pro) in the coding region of the *VSX1* gene to the existing range of identified *VSX1* variations. However, it was predicted that this novel variant may not cause a pathogenic change. Large-scale studies recruiting more patients are required to obtain a deeper understanding of the genetic mechanisms underlying the pathogenesis of keratoconus.

## Abbreviations

PCR-SSCP: Polymerase chain reaction single-strand conformation polymorphism
SNP: Single-nucleotide polymorphism
*VSX1*: Visual system homeobox gene 1
